# Supplementary material for: Novel Driver Strength Index highlights important cancer genes in TCGA PanCanAtlas patients
Source: PeerJ. 2022 Aug 11;10:e13860. doi: 10.7717/peerj.13860 (PMC9375969; doi:10.7717/peerj.13860)
Supplement: Supplemental Information 2 [file peerj-10-13860-s002.zip › COHORTS/genes plots/stage/2021_8_16_14_9_distribution_stage_females_I.pdf]

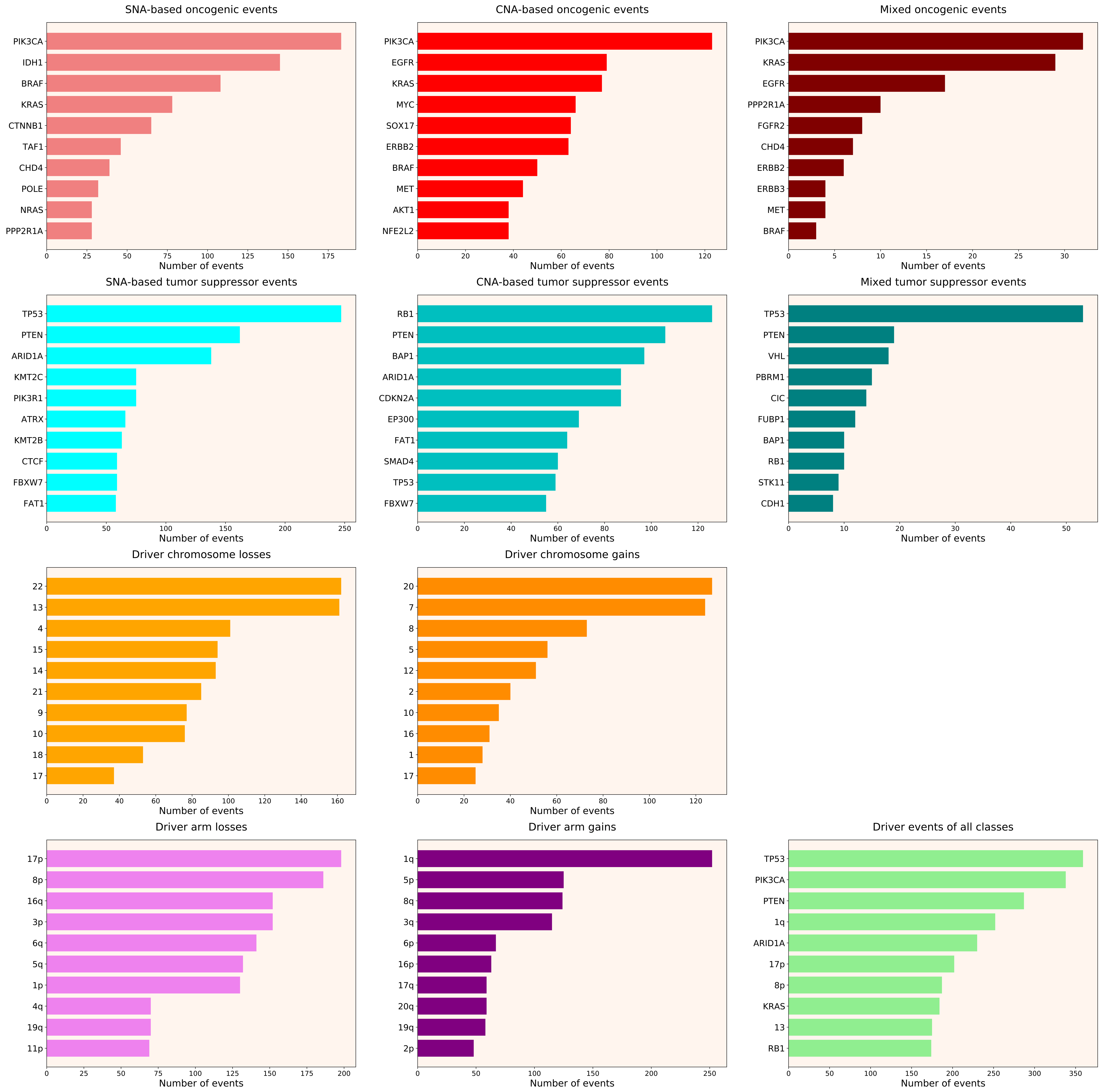
SNA-based oncogenic events
PIK3CA
IDH1
BRAF
KRAS
CTNNB1
TAF1
CHD4
POLE
NRAS
PPP2R1A
Number of events
CNA-based oncogenic events
PIK3CA
EGFR
KRAS
MYC
SOX17
ERBB2
BRAF
MET
AKT1
NFE2L2
Number of events
Mixed oncogenic events
PIK3CA
KRAS
EGFR
PPP2R1A
FGFR2
CHD4
ERBB2
ERBB3
MET
BRAF
Number of events
SNA-based tumor suppressor events
TP53
PTEN
ARID1A
KMT2C
PIK3R1
ATRX
KMT2B
CTCF
FBXW7
FAT1
Number of events
CNA-based tumor suppressor events
RB1
PTEN
BAP1
ARID1A
CDKN2A
EP300
FAT1
SMAD4
TP53
FBXW7
Number of events
Mixed tumor suppressor events
TP53
PTEN
VHL
PBRM1
CIC
FUBP1
BAP1
RB1
STK11
CDH1
Number of events
Driver chromosome losses
22
13
4
15
14
21
9
10
18
17
Number of events
Driver chromosome gains
20
7
8
5
12
2
10
16
1
17
Number of events
Driver arm losses
17p
8p
16q
3p
6q
5q
1p
4q
19q
11p
Number of events
Driver arm gains
1q
5p
8q
3q
6p
16p
17q
20q
19q
2p
Number of events
Driver events of all classes
TP53
PIK3CA
PTEN
1q
ARID1A
17p
8p
KRAS
13
RB1
Number of events
